# PReS-FINAL-2094: Evaluation of the benefits of sequential addition of leflunomide in patients with polyarticular course juvenile idiopathic arthritis failing standard dose methotrexate

**DOI:** 10.1186/1546-0096-11-S2-P106

**Published:** 2013-12-05

**Authors:** PR Chickermane, RP Khubchandani

**Affiliations:** 1 Pediatrics, Jaslok Hospital and Research Center, Mumbai, India

## Introduction

Methotrexate (MTX), the disease modifying antirheumatic drug (DMARD) of first choice in juvenile idiopathic arthritis (JIA), is not effective in 45-50% of patients with polyarticular course JIA. In developed countries, when MTX fails, the next step is to start biologicals. In resource limited settings this step is constrained by prohibitive cost and children failing MTX run the risk of poor disease control or steroid overuse. There is paucity of data on use of other DMARDs or their combinations in such children. MTX and leflunomide (LEF) are known to have differing and complementary actions in modifying the immune response and this combination has been studied in adults. Their combined cost is a fraction of the cost of biologicals.

## Objectives

To evaluate the benefits of addition of LEF in children with polyarticular course JIA, non-responsive to standard dose parenteral MTX.

## Methods

In an observational study, 32 children with polyarticular course JIA (JIA defined by modified ILAR criteria) failing standard dose MTX (up to 15 mg/m2/week sc for at least 3 and up to 6 months) received additional LEF (dosage by body weight). Permitted concomitant drugs included pulse steroids for flares and/or low bridging dose of prednisolone, intra-articular steroids and non-steroidal anti-inflammatory drugs. No other DMARDs were or had been used before enrolment. Patients were assessed once every 8-12 weeks. Parameters recorded at each visit included physician global assessment of disease activity, parent/patient assessment of overall well-being, functional ability, number of joints with active arthritis, number of joints with limited range of motion and laboratory parameters, viz., hemogram, ESR and liver enzymes. The primary efficacy outcome was the ACR Pedi 30 criteria. At the end of follow up, Wallace's criteria were used to determine the percentage of children achieving remission

## Results

25 of the 32 children who followed up for at least 3 months were analysed. The mean follow up duration was 1.61 years (range-0.29 to 3.0 years). At 3 months, 68% of the patients met the ACR Pedi 30 response. 18 of the 21 children (85.7%) showed an ACR Pedi 30 response at 6 months. At 1 year, the percentage of ACR Pedi 30 response was 88.8% with good response rates seen using the ACR Pedi 50 (83.3%), ACR Pedi 70 (61.1%) and ACR Pedi 90 (50%) criteria. Of the 18 children who followed up till the end of the study, 12 (66.6%) met the ACR Pedi 30 criteria and 9(50%) were in clinical remission on medications (off steroids). Adverse effects were observed in 2 children (gastritis and elevated liver enzymes in one each). One child had macrophage activation syndrome temporally related to the introduction of LEF but it is difficult to comment on a causal relationship. Only 2 children among those failing the combination could afford biologicals, thus highlighting the need for such studies. Of the 7 children who were excluded from analysis,3 had developed hepatitis A and 4 had irregular visits.

## Conclusion

Our findings support the further study of the role of this combination in the management of polyarticular course JIA refractory to standard dose MTX, especially in resource challenged settings. The open observational nature of the study is its limitation.

## Disclosure of interest

None declared.

